# Genome-wide analysis of UDP-glycosyltransferase super family in *Brassica rapa* and *Brassica oleracea* reveals its evolutionary history and functional characterization

**DOI:** 10.1186/s12864-017-3844-x

**Published:** 2017-06-23

**Authors:** Jingyin Yu, Fan Hu, Komivi Dossa, Zhaokai Wang, Tao Ke

**Affiliations:** 1 Department of Life Science and Technology, Nanyang Normal University, Wolong Road, Nanyang, 473061 China; 2 Third Institute of Oceanography, State Oceanic Administration, Fujian, 361005 China; 3 Key Laboratory of Biology and Genetic Improvement of Oil Crops, Ministry of Agriculture, Oil Crops Research Institute, the Chinese Academy of Agricultural Sciences, Wuhan, 430062 China

**Keywords:** Glycosyltransferase, Glycosylation, *Brassica*, Tandem duplication, Whole genome duplication, Expression divergence

## Abstract

**Background:**

Glycosyltransferases comprise a highly divergent and polyphyletic multigene family that is involved in widespread modification of plant secondary metabolites in a process called glycosylation. According to conserved domains identified in their amino acid sequences, these glycosyltransferases can be classified into a single UDP-glycosyltransferase (UGT) 1 superfamily.

**Results:**

We performed genome-wide comparative analysis of UGT genes to trace evolutionary history in algae, bryophytes, pteridophytes, and angiosperms; then, we further investigated the expansion mechanisms and function characterization of UGT gene families in *Brassica rapa* and *Brassica oleracea*. Using Hidden Markov Model search, we identified 3, 21, 140, 200, 115, 147, and 147 UGTs in *Chlamydomonas reinhardtii*, *Physcomitrella patens*, *Selaginella moellendorffii*, *Oryza sativa*, *Arabidopsis thaliana*, *B. rapa*, and *B. oleracea*, respectively. Phylogenetic analysis revealed that UGT80 gene family is an ancient gene family, which is shared by all plants and UGT74 gene family is shared by ferns and angiosperms, but the remaining UGT gene families were shared by angiosperms. In dicot lineage, UGTs among three species were classified into three subgroups containing 3, 6, and 12 UGT gene families. Analysis of chromosomal distribution indicates that 98.6 and 71.4% of UGTs were located on *B. rapa* and *B. oleracea* pseudo-molecules, respectively. Expansion mechanism analyses uncovered that whole genome duplication event exerted larger influence than tandem duplication on expansion of UGT gene families in *B. rapa*, and *B. oleracea*. Analysis of selection forces of UGT orthologous gene pairs in *B. rapa*, and *B. oleracea* compared to *A. thaliana* suggested that orthologous genes in *B. rapa*, and *B. oleracea* have undergone negative selection, but there were no significant differences between *A. thaliana* –*B. rapa* and *A. thaliana* –*B. oleracea* lineages. Our comparisons of expression profiling illustrated that UGTs in *B. rapa* performed more discrete expression patterns than these in *B. oleracea* indicating stronger function divergence. Combing with phylogeny and expression analysis, the UGTs in *B. rapa* and *B. oleracea* experienced parallel evolution after they diverged from a common ancestor.

**Conclusion:**

We first traced the evolutionary history of UGT gene families in plants and revealed its evolutionary and functional characterization of UGTs in *B. rapa*, and *B. oleracea*. This study provides novel insights into the evolutionary history and functional divergence of important traits or phenotype-related gene families in plants.

**Electronic supplementary material:**

The online version of this article (doi:10.1186/s12864-017-3844-x) contains supplementary material, which is available to authorized users.

## Background

Glycosylation represents the last step in the biosynthesis of numerous natural compounds, such as terpenes, phenylpropanoids, cyanogenic glucosides, and glucosinolates in plants [1]. Glycosylation can also occur during the terminal synthesis of many other molecules such as glycoproteins, proteoglycans, and hormones. Together with hydroxylation, acylation, and methylation reactions, glycosylation performs a significant function in the diversity and complexity of plant secondary metabolites. Glycosylation is essential for the maintenance of cellular homeostasis through regulating the level, activity, and location of key cellular metabolites [2]. Glycosyl transfer reactions have been highlighted as the most important biotransformation on earth, as in quantitative terms, these reactions account for the assembly and degradation of the bulk of biomass [3]. Moreover, glycosylation is believed to perform an important function in plant defense and stress tolerance [4] because aglycones include plant hormones, secondary metabolites involved in stress and defense responses, and xenobiotics, such as herbicides [2].

The glycosylation process is catalyzed by glycosyltransferase enzymes (GTs), which are highly divergent, polyphyletic, and belong to a multigene family found in all living organisms [5]. GTs are encoded by large families estimated to be 1–2% of the gene products in archaeal, bacterial, and eukaryotic organisms [6]. To date, GTs from diverse species have been classified into 98 families based on similarities of amino acids, substrate specificity, catalytic mechanisms (inversion or retention of anomeric carbon) and the presence of conserved sequence motifs [3, 7]. UDP-dependent glycosyltransferases (UGTs) belong to the largest family (family 1) of the glycosyltransferase superfamily and present distinct but overlapping substrate specificities [5].

For the nomenclature system, UGT genes are named using the UGT Nomenclature Committee conventions: the root symbol UGT, an Arabic number representing the family, a letter to denote the subfamily, and an Arabic number for the individual gene. Families 1–50 are used for animals, 51–70 for yeast, 71–100 for plants, and 101–200 for bacteria [5]. Plant UGTs are thus classified within 30 families between UGT71 and UGT100 [8]. The UGT family has been widely studied in different plant species, as well as animals and humans. In humans, 19 UGT isoforms have been identified, and these UGTs have been classified into two families (UGT1 and UGT2) based on their sequence identity [9]. In the model plant, *Arabidopsis thaliana*, more than 120 UGTs encoding genes have been identified, with five UGTs found to be correlated with flavonoid biosynthetic [10–12]. A total of 253 UGTs were found in *Panax ginseng* transcriptome, and recent works have confirmed the function of UGTs in ginsenoside biosynthesis [13–15]. The genome-wide analysis of UGTs in Flax (*Linum usitatissimum*) led to the identification of 137 UGTs, and subsequent similar works conducted by Sharma et al. (2014) yielded 96 UGTs in Chickpea genome [1, 16]. More recently, 178 UGTs were also found in tea (*Camellia sinensis*), and 30 candidate genes were validated for their function in the biosynthesis of astringent taste compounds [17].

At present, the genome release of representative plant species provided valuable genomic materials for scientists to investigate the evolutionary and functional characterization of gene families related to important traits and phenotypes. *A. thaliana* was an important model plant with relatively complete genomic data [18]. *B. rapa* and *B. oleracea* are important diploid species in genus *Brassica* with finished genome projects and released genome data [19, 20]. A previous study uncovered that the *Brassica* ancestor diverged from a common ancestor with *A. thaliana* approximately 20 million years ago (Mya); subsequently, the *Brassica* ancestor experienced whole genome triplication (WGT) event approximately 16 Mya. Then, the *Brassica* ancestor diverged to form the modern *B. rapa* and *B. oleracea* about 3.75 Mya [21–24]. The WGT event brought the increase in genomic materials in *Brassica* species, providing an excellent evolutionary model to investigate the expansion of gene families. In addition to genome duplication, tandem duplication (TD) event is another important mechanism that induces the increase in the members of gene families, leading to expansion of gene families [25].

In the present study, we selected several genome-sequenced plant species to trace the evolutionary history of the UGT gene family and investigate the appearance of UGT gene family in different evolutionary stages of plant species. These species included the representative plant species from lower unicellular algae to higher angiosperms and also released their genome sequences, which brought convenience for researchers to study UGT gene family based on genome wide. For example, *Chlamydomonas reinhardtii* is an ancient unicellular green alga, which was diverged from land plants over 1 billion years ago [26]. The model moss *Physcomitrella patens* is a bryophyte (land plants), which is separated by more than 400 million years from flowering plants [27]. *Selaginella moellendorffii* is a non-seed vascular plant that provide a useful resource for identifying genes that may have been important in the early evolution of developmental and metabolic processes that are unique to vascular plants [28]. A draft sequence of *Oryza sativa* L. ssp. Japonica genome has been reported, which provides a foundation for the improvement of cereals [29]. Except for the above plant species, *A. thaliana*, *B. rapa*, and *B. oleracea* were included. These seven plant species demonstrate clear phylogenetic relationship, which provided clear evolutionary paths for the UGT gene families in plants. After identifying the members of UGT gene families among seven plant species, we investigated ancient UGT genes and UGT gene families sharing different plants according to the phylogenetic relationship among seven species. Furthermore, we systematically studied the expansion mechanism and functional characterization of UGT gene families in *A. thaliana* and two *Brassica* species owing to their good evolutionary model. For the two *Brassica* species, we detected chromosomal localization of UGT genes and the evolutionary relationship of the members of UGT super gene family in *B. rapa*, and *B. oleracea*. To explore expansion mechanism of UGT gene family in *Brassica*, we also studied the influences of WGT and TD events on the expansion of UGT super gene family. According to the syntenic relationships in *B. rapa* and *B. oleracea* compared with *A. thaliana*, we investigated the selection pressures of UGT orthologous gene pairs between *A. thaliana*–*B. rapa* and *A. thaliana*–*B. oleracea* lineages. Furthermore, we analyzed the functional divergence of the members of UGT gene family by using the expression values of UGT genes in *B. rapa*, and *B. oleracea*.

## Methods

### Data sources

Genomic data of *C. reinhardtii*, *P. patens*, and *S. moellendorffii* were obtained from Phytozome (the Plant Comparative Genomics portal of the Department of Energy’s Joint Genome Institute; http://genome.jgi-psf.org/) [30]. Genomic and assembly data of *O. sativa* L. *ssp.* Japonica, *A. thaliana*, *B. rapa*, and *B. oleracea* were obtained from RGAP (http://rice.plantbiology.msu.edu) [31], TAIR10 (http://www.arabidopsis.org) [32], BRAD (http://brassicadb.org/brad) [33], and Bolbase (http://ocri-genomics.org/bolbase) [34]. The putative tandem-duplicated genes of *A. thaliana*, *B. rapa*, and *B. oleracea* were retrieved from PTGBase (http://ocri-genomics.org/PTGBase) [35]. The Hidden Markov Model (HMM) profile of UDPGT domain (PF00201) was retrieved from Pfam 29.0 (http://pfam.xfam.org/) [36]. The expression profile data of *B. rapa* and *B. oleracea* were downloaded from the Gene Expression Omnibus (GEO) database with accession numbers GSE43245 and GSE42891, respectively [20, 37, 38].

### Identification of UGT genes

The putative UGT genes of *C. reinhardtii*, *P. patens*, *S. moellendorffii*, *O. sativa*, *A. thaliana*, *B. rapa*, and *B. oleracea* were identified by HMMER V3.0 program with “trusted cutoff” as threshold [39]. Using the putative UGT genes, high conserved UGT protein sequences were selected to implement multiple sequence alignments through CLUSTALW [40]. Then, the highly conserved UGT protein sequences were used to construct species-specific HMM profile by using the “hmmbuild” module by HMMER V3.0 program. Using the species-specific HMM profile of UGTs, the final UGT gene sets were classified from the genomic data of the above seven plant species through HMMER V3.0 program with stringent parameters. Finally, InterPro was used to check the validation of final UGT genes [41].

### Distribution of UGT genes on pseudo-molecular chromosomes

According to the general feature format (GFF) files of UGT genes from *Brassica* species-specific genome database, 18 in-house Perl and Python scripts were used to draw the graphics of UGT genes on pseudo-molecular chromosomes with scalable vector graphic module [42].

### Phylogenetic analysis of UGT genes

Following the final UGT genes datasets, UGT protein sequences were used to implement multiple sequence alignment using the program CLUSTALW with default options [40]. Then, the output files were used to construct phylogenetic trees using Maximum Likelihood (ML) method in FastTree with 1000 replications [43].

### Syntenic analysis in *Brassica* species compared to *A. thaliana*

MCScanX toolkit was employed to identify putative homologous chromosomal regions between *Brassica* genomes and *A. thaliana* genomes with the parameters (*e* = 1 × 10^−20^, *u* = 1, and *s* = 5. Parameter of *s* = 5) [44]. According to the syntenic analysis procedures using MCScan algorithm, high similarity genes were recognized as anchors to align homologous chromosomal regions between multiple genomes or subgenomes, which can help us to identify orthologous gene pairs in *Brassica* species compared with *A. thaliana* genomes.

### Expression profiling of UGT genes in *Brassica*

The expression values for each UGT gene were calculated by fragments per kilobase of the exon model per million mapped reads by using the RNA-seq data of *B. rapa* and *B. oleracea* from the GEO database [45]. The spearman rank correlation was used to calculate the distance matrix between the gene expression data of UGTs in *B. rapa* and *B. oleracea*. Further, the complete linkage clustering was used for hierarchical clustering of UGT genes in *B. rapa* and *B. oleracea*. Expression heat maps of UGT genes in *B. rapa* and *B. oleracea* were generated by R language packages.

### Selection pressures of UGT gene pairs between *Brassica* species and *A. thaliana*

The ratios of the rates of nonsynonymous to synonymous substitutions (*K*_a_/*K*_s_) of UGT gene pairs between *Brassica* species and *A. thaliana* were calculated to estimate selection modes by using PAML software [46]. The *K*_a_/*K*_s_ ratios greater than 1, less than 1, and equal to 1 represent positive selection, negative selection, and neutral selection, respectively.

### Statistical tests

The significance of the difference in the *K*_a_/*K*_s_ values of UGT gene pairs between two samples was evaluated by using Mann-Whitney U test with R language packages.

## Results

### Identification and characterization of UGT genes in plants

Based on the latest version of genome-sequenced plant genomes, we used HMMER 3.0 program to search amino acid sequences in *C. reinhardtii*, *P. patens*, *S. moellendorffii*, *O. sativa* L. *ssp.* Japonica, *A. thaliana*, *B. rapa*, and *B. oleracea* based on the HMM profile of UGT-conserved domains to identify UGT genes among the seven plant species. After curation of UGT gene datasets, we retrieved three UGT genes in *C. reinhardtii*, 21 in *P. patens*, 140 in *S. moellendorffii*, 200 in *O. sativa* L. *ssp.* Japonica, 115 in *A. thaliana*, and 147 in each of *B. rapa* and *B. oleracea.* The seven plants were distributed in algae, bryophytes, pteridophytes, monocots, and dicots. From the numbers of UGT gene family in different species, we found that the members of UGT gene family exhibit an increased tendency from lower plants to higher plants, and plant species of monocots may contain more UGT genes than dicots. According to a comparative analysis of UGT genes between *A. thaliana* and *Brassica* species, *Brassica* species possess almost 1.28 times the UGT genes of *A. thaliana* in dicots. According to the datasets of UGT genes and the phylogenetic relationship among the seven species, we can investigate the appearance of UGT gene family in different evolutionary stages of plants.

### Phylogenetic analysis of UGT super gene family in plant

**Fig. 1:**
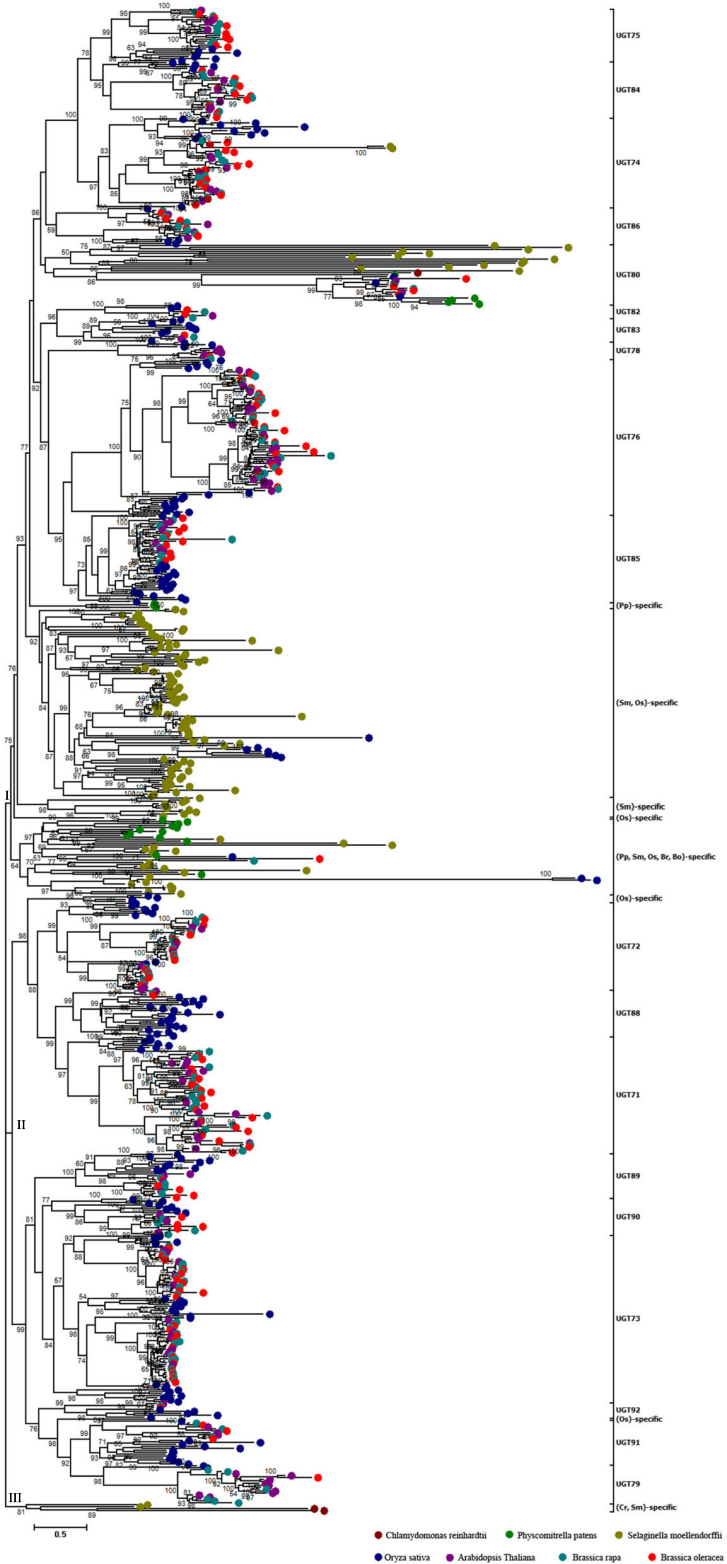
Phylogenetic relationship of UGT genes in genome sequenced plants. The Maximum Likelihood tree was constructed by FastTree software with 1000 replications. Maroon, green, olive, navy, purple, teal, and red solid circles represent the UGT genes in *C. reinhardtii*, *P. patens*, *S. moellendorffii*, *O. sativa*, *A. thaliana*, *B. rapa*, and *B. oleracea*, respectively

The previous study explored UGT genes, which were distributed into 21 UGT gene families, in *A. thaliana*. Based on the phylogenetic relationship among UGT genes, the members of the same UGT gene families will be clustered together. According to unique signature motifs or domains from alignments of protein sequences, 772 UGT genes in seven plant species were used to construct phylogenetic tree for detecting the phylogenetic relationship by ML method with FastTree software [43]. All UGT genes among the seven species were clustered into three major subgroups: I, II, and III subgroups (Fig. 1). The I subgroup contained 455 UGT genes, which were distributed into 10 UGT gene families and five species-specific clusters, representing 58.94% of total UGT genes among seven species (Table 1). Among the 10 UGT gene families, eight UGT gene families were shared by *O. sativa*, *A. thaliana*, *B. rapa*, and *B. oleracea*, indicating that the UGT gene families exist in angiosperms. However, the members of UGT80 gene family was detected in algae, bryophytes, ferns and angiosperms, suggesting this UGT gene family is the ancestral UGT gene family in plants. The members of UGT74 gene family were identified in *S. moellendorffii*, *O. sativa*, *A. thaliana*, *B. rapa*, and *B. oleracea*, meaning that this UGT gene family is shared by ferns, monocots, and dicots. In addition, five species-specific UGT gene clusters were classified in subgroup I, with three single species-specific UGT clusters distributed among *P. patens*, *S. moellendorffii*, and *O. sativa*. The remaining two UGT clusters were composite species-specific UGT clusters. One UGT cluster contained 97 UGT genes that are shared by *S. moellendorffii* and *O. sativa*, and the other cluster contained 39 UGT genes, which were distributed in *P. patens*, *S. moellendorffii*, *O. sativa*, *B. rapa*, and *B. oleracea*. The former indicates that the 97 UGT genes may be specific UGT genes in ferns and monocots, whereas the latter indicates that these UGT genes are shared by bryophytes, fern, and angiosperm; however, *A. thaliana* is missing members in this cluster. In subgroup II, we identified 313 UGT genes representing 40.54% of total UGT genes in seven species. These UGT genes were distributed into nine UGT gene families and one species-specific UGT cluster. Among these UGT gene families, the members of nine UGT gene families were distributed in *O. sativa*, *A. thaliana*, *B. rapa*, and *B. oleracea*, suggesting that these UGT gene families were shared by monocots and dicots (or angiosperm). Except for UGT gene families, only one species-specific UGT cluster was detected in this subgroup, and this species-specific UGT cluster contains five UGT genes in *O. sativa.* The III subgroup only contained one species-specific UGT cluster, which includes four UGT genes shared by *C. reinhardtii* and *S. moellendorffii*.

**Table 1:** Summary of UGT genes in genome sequenced plants

Type	Cluster or Gene family	*C. reinhardtii*	*P. patens*	*S. moellendorffii*	*O. sativa*	*A.thaliana*	*B. rapa*	*B. oleracea*
I	UGT74	/	/	2	12	7	11	14
	UGT75	/	/	/	7	4	7	9
	UGT76	/	/	/	17	21	20	22
	UGT78	/	/	/	3	4	1	1
	UGT80	1	5	14	3	2	2	4
	UGT82	/	/	/	2	1	2	2
	UGT83	/	/	/	9	1	1	1
	UGT84	/	/	/	4	6	9	10
	UGT85	/	/	/	21	6	9	9
	UGT86	/	/	/	4	4	6	5
	(Os)-specific	/	/	/	1	/	/	/
	(Pp)-specific	/	3	/	/	/	/	/
	(Pp,Sm,Os,Br,Bo)-specific	/	13	21	3	/	1	1
	(Sm)-specific	/	/	10	/	/	/	/
	(Sm,Os)-specific	/	/	90	7	/	/	/
II	UGT71	/	/	/	7	14	19	20
	UGT72	/	/	/	9	9	14	13
	UGT73	/	/	/	26	13	26	21
	UGT79	/	/	/	2	11	6	1
	UGT88	/	/	/	21	1	1	1
	UGT89	/	/	/	10	4	4	5
	UGT90	/	/	/	8	3	4	4
	UGT91	/	/	/	14	3	3	3
	UGT92	/	/	/	5	1	1	1
	(Os)-specific	/	/	/	5	/	/	/
III	(Cr, Sm)-specific	2	/	2	/	/	/	/

Among the seven species, phylogenetic analysis revealed that several UGT gene families or clusters were shared by different plant species, which can reflect putative emergence evolutionary periods of different UGT gene families or clusters in the phylogenetic tree. Furthermore, analysis of expansion mechanism and functional characterization of UGT gene families will be investigated comprehensively in *A. thaliana*, *B. rapa*, and *B. oleracea* owing to their excellent phylogenetic model in dicots.

### Chromosomal localization of UGT genes in *B. rapa, and B. oleracea*

To detect detailed information on UGT genes in *Brassica*, we investigated the chromosomal localization of UGT genes in *Brassica* species according to gene annotation files retrieved from public genomic databases (Additional file 1). After curation, 145 UGT genes in *B. rapa* and 105 UGT genes in *B. oleracea* were located on pseudo-molecules/chromosomes, which represent 98.6 and 71.4% of the total UGT genes in *B. rapa* and *B. oleracea*, respectively. The chromosomal localization of UGT genes is uneven in *Brassica* genomes. In the *B. rapa* genome, 29 UGT genes are located on the A05 chromosome, which contains the most number of UGT genes, and A07 chromosome only contains six UGT genes. In the *B. oleracea* genome, the chromosome containing the most number of UGT genes (20 UGT genes) is C03 chromosome, and C02 chromosome only contained three UGT genes and contains the least number of UGT genes compared with other chromosomes.

Gene cluster is defined as two or more genes falling within eight open reading frames on the same chromosome, which is used to describe relative positions on chromosomes between adjacent genes. Previous reports have described the relative position between adjacent disease resistance genes (R genes) in *A. thaliana*, which provided an intuitive and clear impression for researchers to understand the organization of R genes on the chromosome [47, 48]. For the UGT superfamily, gene cluster is also used to describe the relative positions of UGT genes in the *Brassica* species. According to the definition of gene clusters, 29 UGT gene clusters covering from two to six UGT genes were detected in *B. rapa*. All UGT gene clusters in *B. rapa* contains 81 UGT genes, which represent 55.86% of all UGT genes located on chromosomes. The remaining 64 UGT genes are anchored on chromosomes as singletons. In *B. oleracea*, 53 UGT genes are distributed in 19 UGT gene clusters, representing 50.48% of total UGT genes located on chromosomes in *B. oleracea*, with the remaining UGT genes as singletons.

### Evolutionary relationships of UGT genes in *B. rapa, and B. oleracea*

**Fig. 2:**
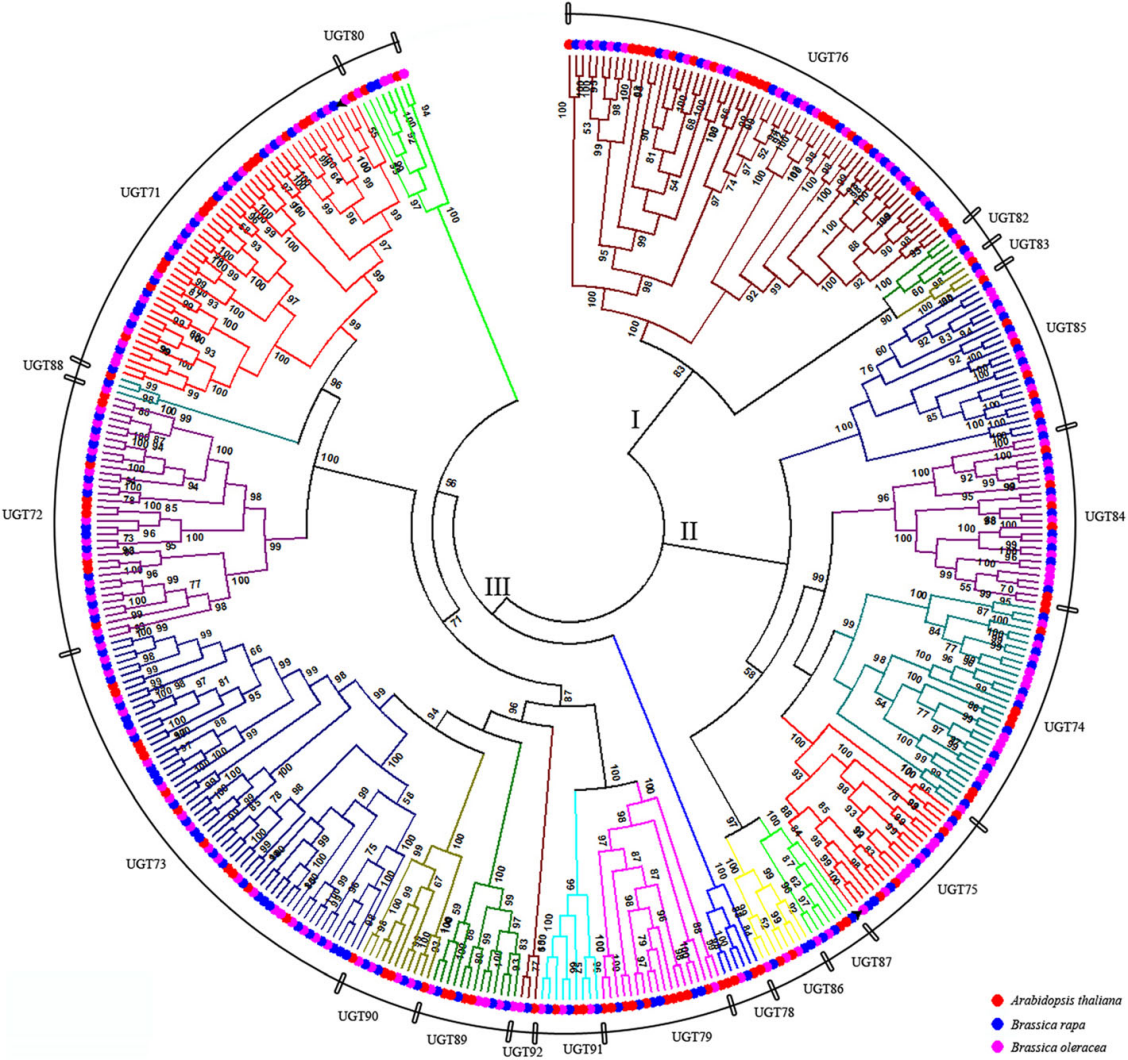
Phylogenetic analysis of UGT gene families among *A. thaliana*, *B. rapa*, and *B. oleracea*. The Maximum Likelihood tree was constructed by FastTree software with 1000 replications. I, II, and III represent different subgroups. Each UGT gene family is shown by different colors. Red, blue, and pink solid circles represent the UGT genes in *A. thaliana*, *B. rapa*, and *B. oleracea*, respectively

*A. thaliana* have diverged from a common ancestor with a *Brassica* ancestor, and then the *Brassica* ancestor experienced a WGT event about 18 Mya [49]. After the WGT event, the *Brassica* ancestor diverged to form the modern *B. rapa* and *B. oleracea*. To detect evolutionary relationship of UGT genes in *Brassica*, we employed 409 UGT genes in *A. thaliana*, *B. rapa*, and *B. oleracea* to investigate their phylogenetic relationship and further confirm the members of UGT gene families in *Brassica* species (Fig. 2).

Through the phylogenetic analysis of total UGT genes among three species, the phylogenetic tree was divided into three different subgroups, namely, subgroups I, II, and III. Subgroup I contains three UGT gene families, which covered 71 UGT genes among three species (Table 2). Subgroup II covers 117 UGT genes, which are distributed in six UGT gene families and one *B. oleracea* -specific UGT cluster. Compared with the above two subgroups, Subgroup III covers the most number of UGT genes (221 UGTs) representing 54.03% of total UGT genes among three species. This subgroup contained 12 UGT gene families and one *B. rapa*-specific UGT cluster. From the above phylogenetic analysis of UGT genes among seven species, one composite species-specific UGT cluster contains 39 UGT genes, which are distributed in *P. patens* (13 UGTs), *S. moellendorffii* (21 UGTs), *O. sativa* (3 UGTs), *B. rapa* (1 UGTs, Bra031561), and *B. oleracea* (1 UGTs, Bol040997) in Subgroup I, and *A. thaliana* is missing its members in this cluster. This result suggests that the UGT genes in *B. rapa* and *B. oleracea* were *Brassica*-specific UGT genes. Through phylogenetic analysis of UGT genes among *A. thaliana*, *B. rapa*, and *B. oleracea*, *Brassica-*specific UGT gene (Bra031561) in *B. rapa* is clustered with the members of UGT71 gene family, and the other *Brassica-*specific UGT gene (Bol040997) in *B. oleracea* is clustered with the members of UGT75 gene family. This result indicates that the two *Brassica-*specific UGT genes present closer relative relationships with the members of UGT71 or UGT75 gene families, despite being *Brassica*-specific UGT genes.

**Table 2:** Statistics of UGT genes in UGT gene families among *A. thaliana*, *B. rapa*, and *B. oleracea*

Type	Family	*A. thaliana*	*B. rapa*	*B. oleracea*
I	UGT76	21	20	22
	UGT82	1	2	2
	UGT83	1	1	1
II	UGT74	7	11	14
	UGT75	4	7	9
	*B.oleracea*_specific	/	/	1
	UGT84	6	9	10
	UGT85	6	9	9
	UGT86	2	3	3
	UGT87	2	3	2
III	UGT71	14	19	20
	*B. rapa*_specific	/	1	/
	UGT72	9	14	13
	UGT73	13	26	21
	UGT78	4	1	1
	UGT79	11	6	1
	UGT80	2	2	4
	UGT88	1	1	1
	UGT89	4	4	5
	UGT90	3	4	4
	UGT91	3	3	3
	UGT92	1	1	1

### Analysis of WGT events for UGT genes in *B. rapa, and B. oleracea*

The ancestor of diploid *Brassica* species experienced a WGT event after splitting from a common ancestor with *A. thaliana*, meaning that one genomic region in *A. thaliana* has retained three copies of orthologous genomic regions in *B. rapa* and *B. oleracea* genomes. According to the difference of gene density, the three orthologous genomic regions in *B. rapa* or *B. oleracea* were classified into three sub-genomes, namely, MF1 (medium fractionated), MF2 (most fractionated), and LF (least fractionated) [19]. Based on the syntenic relationship datasets among *A. thaliana* and *Brassica* species, 74 *A. thaliana* UGT genes were detected corresponding orthologous genes in the three sub-genomes of *B. rapa* or *B. oleracea* (Additional file 2). Through syntenic analysis between *A. thaliana* and *B. rapa*, we detected 65 UGT genes in *A. thaliana* that are orthologous UGT genes in the three sub-genomes of *B. rapa*. Among 65 UGT genes in *A. thaliana*, 39, 25, and two UGT genes were one-, two- and three-copy orthologous UGT gene(s) retained in corresponding syntenic regions in the three sub-genomes of *B. rapa*, referring to 94 UGT genes representing 63.95% of the total UGT genes in *B. rapa*. According to syntenic relationship between *A. thaliana* and *B. oleracea*, 65 *A. thaliana* UGT genes include orthologous UGT genes in the three sub-genomes of *B. oleracea*. Among 65 UGT genes in *A. thaliana*, 45, 15, and five UGT genes showed one-, two- and three-copy orthologous UGT gene(s) retained in the corresponding syntenic regions in the three sub-genomes of *B. oleracea*. Therefore, 65 UGT genes in *A. thaliana* can be obtained from 90 orthologous UGT genes in *B. oleracea*, representing 61.22% of total UGT genes in *B. oleracea*.

### Selection forces of UGT orthologous gene pairs in *B. rapa, and B. oleracea* compared with *A. thaliana*

**Fig. 3:**
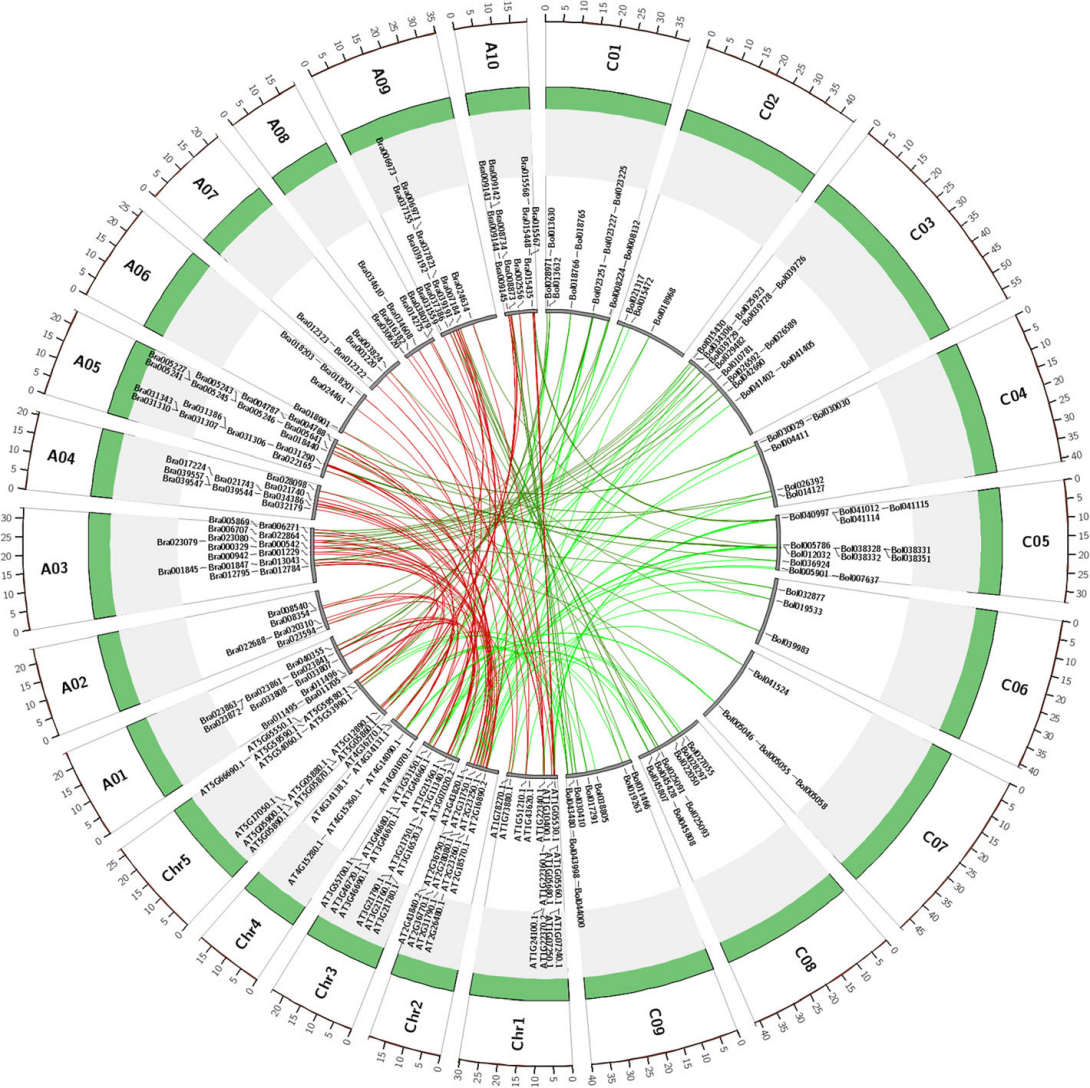
Circos diagram of UGT orthologous genes among *A. thaliana*, *B. rapa*, and *B. oleracea*. Green bars represent pseudo-chromosomes in three species. A01–A10 represent pseudo-chromosomes in *B. rapa*. C01–C09 represent pseudo-chromosomes in *B. oleracea*. Chr1–Chr5 represent pseudo-chromosomes in *A. thaliana*. Red lines represent UGT orthologous gene pairs between *A. thaliana* and *B. rapa*. Blue lines represent UGT orthologous gene pairs between *B. rapa* and *B. oleracea*. Green lines represent UGT orthologous gene pairs between *A. thaliana* and *B. oleracea*

Selection force is usually measured by calculating the ratio between the number of nonsynonymous substitutions per nonsynonymous site (*K*_a_) and the number of synonymous substitution per synonymous site (*K*_s_). To detect whether UGT orthologous gene pairs in *Brassica* species compared to *A. thaliana* experienced different selective forces, we calculated the *K*_a_/*K*_s_ values associated with terminal branches to measure different selection forces by using PAML software [46]. Through the syntenic relationship of UGT genes in *A. thaliana* and *Brassica* species, we obtained 74 *A. thaliana* UGT genes with orthologous genes in *Brassica* species. Considering that the 74 *A. thaliana* UGT genes exhibit different retention or loss patterns of orthologous genes among different sub-genomes in *B. rapa* and *B. oleracea*, we finally found that 65 *A. thaliana* UGT genes present 94 orthologous UGT genes in *B. rapa*, whereas 65 partially different *A. thaliana* UGT genes present 90 orthologous UGT genes in *B. oleracea*. According to the syntenic regions in *B. rapa* and *B. oleracea* compared with *A. thaliana*, we extracted 75 UGT orthologous gene pairs between *B. rapa* and *B. oleracea* (Fig. 3). These syntenic orthologous UGT gene pairs in *Brassica* species compared with *A. thaliana* offered us a rich genomic resource to investigate selection forces of UGT orthologous gene pairs between *A. thaliana*–*B. rapa* and *A. thaliana*–*B. oleracea* lineages. After curation of orthologous gene pairs in *Brassica* species compared with *A. thaliana*, 94 and 90 orthologous UGT gene pairs in *A. thaliana*–*B. rapa* and *A. thaliana–B. oleracea* lineages, respectively, were used to detect the differences of selection forces of UGT orthologous gene pairs of *B. rapa* and *B. oleracea* compared with *A. thaliana*. From the comparison of the ratios of UGT orthologous gene pairs among three species, the mean *K*_a_/*K*_s_ ratio of 94 UGT orthologous genes in *B. rapa* compared with *A. thaliana* was 0.2408, which is slightly greater than that (0.2398) of 90 UGT orthologous gene pairs in *B. oleracea* compared with *A. thaliana*. The *K*_a_/*K*_s_ ratios of UGT orthologous gene pairs in *B. rapa* and *B. oleracea* compared with *A. thaliana* were less than 1, indicating that the UGT orthologous gene pairs experienced negative selection in the process of species evolution. The *K*_a_/*K*_s_ ratios of UGT orthologous gene pairs in *B. rapa* and *B. oleracea* compared with *A. thaliana* were found to show no significant differences through statistics analysis by Mann-Whitney U-test (*P*-value = 0.8943 > 0.05, Mann-Whitney U-test).

### Analysis of TD events for UGT genes in *B. rapa, and B. oleracea*

A TD event performs an important function in the expansion of gene families and can produce tandem repeat genes as clusters [50]. The putative tandem-duplicated genes of *A. thaliana*, *B. rapa*, and *B. oleracea* were downloaded from PTGBase (http://ocri-genomics.org/PTGBase/) [35]. After curation, we obtained 63, 67, and 49 tandem-duplicated genes belonging to UGT genes and representing 54.78, 45.58, and 33.33% of total UGT genes in *A. thaliana*, *B. rapa*, and *B. oleracea*, respectively (Additional file 3). For *A. thaliana*, 63 UGT tandem-duplicated genes were distributed in 24 tandem arrays of two to six UGT genes, which belong to the members of 10 UGT gene families. Among of 63 UGT tandem-duplicated genes in *A. thaliana*, 15 UGT tandem-duplicated genes are distributed in the UGT76 gene family, meaning that the UGT76 gene family experienced greater influence of the TD event compared with other UGT gene families. In *B. rapa*, 67 UGT tandem-duplicated genes are distributed in 26 tandem arrays of two to six UGT genes, which refer to 10 UGT gene families. After comparative analysis, 19 UGT tandem-duplicated genes were classified into the UGT73 gene family, which contains the most UGT tandem-duplicated genes in *B. rapa*. In *B. oleracea*, 49 UGT tandem-duplicated genes are distributed in nine UGT gene families with 19 tandem arrays of two to seven UGT genes. The UGT73 gene family includes 11 UGT tandem-duplicated genes, which contain the most number of UGT tandem-duplicated genes in *B. oleracea*. These results suggest that the UGT73 gene family experienced greater influence of the TD event compared with the other UGT gene families in the genus *Brassica*.

### Generation time of TD event for UGT genes in *B. rapa, and B. oleracea*

**Fig. 4:**
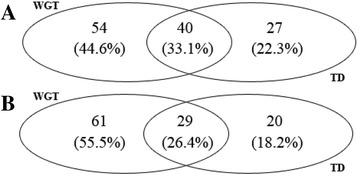
Venn diagrams of UGT genes from whole genome triplication and tandem duplication in *B. rapa* and *B. oleracea*. **a** Venn diagram of UGT genes in *B. rapa*. **b** Venn diagram of UGT genes in *B. oleracea*. WGT represents whole genome triplication. TD represents tandem duplication. The numbers in the Venn diagram represent UGT genes in different parts. The numbers of percentage represent the percentage between UGT genes in different parts and the total UGT genes of three different parts

By combining WGT with TD events in *Brassica* species, we detected that certain UGT genes were generated not only from a WGT event but also a TD event, meaning that these UGT genes were distributed into syntenic genomic regions in *Brassica* species compared with *A. thaliana* as tandem arrays. These results suggest that these UGT tandem-duplicated genes existed before divergence between *A. thaliana* and the *Brassica* ancestor from a common ancestor. Therefore, these UGT genes were ancient tandem-duplicated genes from the common ancestor of *A. thaliana* and *Brassica* species because all UGT genes on syntenic genomic regions in *Brassica* species possess corresponding UGT orthologous genes in *A. thaliana*. Furthermore, if the UGT genes were shown as tandem arrays in *Brassica* species, the corresponding tandem array in *A. thaliana* would exist on *A. thaliana*-conserved genomic regions. Therefore, these tandem arrays of UGT genes in *A. thaliana* and *Brassica* species were inherited from their common ancestor. Among 94 UGT genes from WGT event in *B. rapa*, 40 UGT genes are tandem-duplicated genes, representing 59.7% of total tandem-duplicated genes (67 UGT genes) in *B. rapa* (Fig. 4a). These 40 UGT genes are ancient tandem-duplicated genes, which are inherited from the common ancestor of *A. thaliana* and *B. rapa*; these UGT genes are co-retained genes in *A. thaliana* and *B. rapa* owing to the syntenic relationship between the species. In the *B. oleracea* genome, 29 of 90 UGT genes from WGT event are tandem-duplicated genes, which represent 59.18% of the total tandem-duplicated genes (49 UGT genes) in *B. oleracea* (Fig. 4b). Similar to the 40 UGT genes in *B. rapa*, these 29 UGT genes are ancient tandem-duplicated genes and co-retained UGT genes in the *A. thaliana*–*B. rapa* lineage.

After curation of ancient UGT tandem-duplicated genes, we retrieved 13 and 12 complete tandem arrays referring to 31 and 24 UGT tandem-duplicated genes in *B. rapa* and *B. oleracea*, respectively (Table 3). The remaining nine and five UGT tandem-duplicated genes in *B. rapa* and *B. oleracea*, respectively, belongs to incomplete tandem arrays, but these genes were generated before the *Brassica* ancestor split from a common ancestor with *A. thaliana.* Except for ancient UGT tandem-duplicated genes in *Brassica* species, 27 and 20 present UGT tandem-duplicated genes are reserved in *B. rapa* and *B. oleracea*, respectively. For the present UGT genes, we identified seven and five complete UGT tandem arrays including 14 and 10 UGT genes in *B. rapa* and *B. oleracea*, respectively. These seven and five UGT tandem arrays, which were specific UGT tandem arrays in *B. rapa* and *B. oleracea*, respectively, were generated after divergence between *A. thaliana* and the *Brassica* ancestor from a common ancestor. After excluding present UGT tandem arrays in *Brassica* species, 13 and 10 present UGT tandem-duplicated genes belonging to incomplete tandem arrays remained. From comparative analysis of ancient and present UGT tandem-duplicated genes in incomplete tandem arrays, we found that the tandem-duplicated genes constituted complete tandem arrays in *Brassica* species. These results suggest that the ancient tandem-duplicated genes in incomplete tandem arrays on WGT regions experienced the TD event and formed the present tandem-duplicated genes in *Brassica* species.

**Table 3:** Ancient tandem arrays of UGT genes in *Brassica* species compared with *A. thaliana*

Family	AGI	*Brassica rapa*			*Brassica oleracea*		
		BraLF	BraMF1	BraMF2	BolLF	BolMF1	BolMF2
UGT71	AT1G07240.1	Bra015567	NA	NA	Bol041114	NA	NA
UGT71	AT1G07260.1	Bra015568	NA	NA	Bol041115	NA	NA
UGT71	AT3G21750.1	Bra031306	NA	NA	Bol038332	NA	NA
UGT71	AT3G21760.1	Bra031307	NA	NA	Bol038331	NA	NA
UGT73	AT2G36750.1	Bra005246	NA	NA	NA	NA	NA
UGT73	AT2G36770.1	Bra005245	NA	Bra023080	NA	NA	Bol039728
UGT73	AT2G36780.1	Bra005243	NA	NA	Bol001666	NA	Bol039729
UGT73	AT2G36800.1	Bra005241	NA	Bra023079	Bol001668	NA	NA
UGT73	AT3G53150.1	Bra006971	NA	NA	NA	NA	NA
UGT73	AT3G53160.1	Bra006973	NA	NA	NA	NA	NA
UGT73	AT4G34131.1	Bra011495	NA	Bra034608	Bol013630	NA	Bol014271
UGT73	AT4G34138.1	Bra011496	NA	Bra034610	Bol013632	NA	Bol014269
UGT74	AT2G43820.1	Bra004787	NA	NA	Bol030029	NA	NA
UGT74	AT2G43840.2	Bra004788	NA	NA	Bol030030	NA	NA
UGT76	AT3G46670.1	NA	Bra033808	NA	NA	Bol018766	NA
UGT76	AT3G46680.1	NA	NA	NA	NA	Bol018765	NA
UGT76	AT3G46690.1	NA	Bra033807	NA	NA	NA	NA
UGT76	AT5G05860.1	Bra009142	NA	NA	Bol044004	NA	NA
UGT76	AT5G05870.1	Bra009143	NA	NA	Bol044003	NA	NA
UGT76	AT5G05880.1	Bra009144	NA	NA	Bol043998	NA	NA
UGT76	AT5G05890.1	NA	NA	NA	Bol044000	NA	NA
UGT76	AT5G05900.1	Bra009145	NA	NA	NA	NA	NA
UGT84	AT2G23250.1	NA	Bra039191	NA	NA	NA	NA
UGT84	AT2G23260.1	NA	Bra039192	NA	NA	NA	NA
UGT84	AT4G15490.1	NA	NA	NA	Bol000782	NA	NA
UGT84	AT4G15500.1	NA	NA	NA	Bol000784	NA	NA
UGT85	AT1G22340.1	Bra031386	NA	NA	NA	NA	NA
UGT85	AT1G22360.1	Bra031387	NA	NA	NA	NA	NA
UGT85	AT1G22370.2	Bra031388	NA	NA	NA	NA	NA
UGT85	AT1G22380.1	NA	Bra012322	NA	NA	Bol041988	NA
UGT85	AT1G22400.1	NA	Bra012323	NA	NA	Bol041987	NA

*NA* not yet assigned

From the comparative analysis of UGT genes generated from WGT and TD events, we found that UGT tandem-duplicated genes in *Brassica* species were composed of three parts: (i) the ancient UGT tandem-duplicated genes in complete tandem arrays, (ii) the combined UGT tandem-duplicated genes between ancient and present tandem-duplicated genes in incomplete tandem arrays, and (iii) specific UGT tandem-duplicated genes in complete tandem arrays.

### Retention or loss of the members of UGT gene families in *Brassica* species compared with *A. thaliana*

**Fig. 5:**
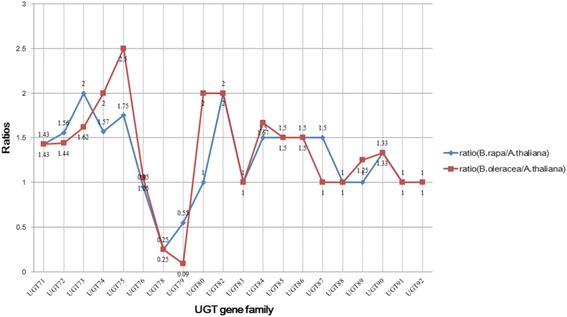
Comparison of the ratios between the numbers of UGT genes in *B. rapa* or *B. oleracea* and the number of UGT genes in *A. thaliana*. The blue line represents the ratios between the number of UGT genes in *B. rapa* and the number of UGT genes in *A. thaliana*. Red line represents the ratios between the number of UGT genes in *B. oleracea* and the number of UGT genes in *A. thaliana*

The WGT event enriches the evolutionary history of the genus *Brassica* and provided abundant genomic materials, which offered a considerable opportunity for scientists to study the retention or loss of the members of UGT gene families in *Brassica* species. On the basis of the conserved domains of UGT genes, we have identified 147 UGT genes in each of *B. rapa* and *B. oleracea* possessing almost 1.28 times the number of UGT genes in *A. thaliana.* For each UGT gene family, we employed the ratio between the numbers of UGT genes in *B. rapa* or *B. oleracea* and the numbers of UGT genes in *A. thaliana* to evaluate the retention or loss of the members of UGT gene family in *Brassica* species compared with *A. thaliana* (Fig. 5). From the comparison of UGT gene families between *B. rapa* and *A. thaliana*, 11 UGT gene families showed the increase in the members of UGT gene families compared with *A. thaliana*, suggesting the expansion of 11 UGT gene families in *B. rapa*. For the rest of the UGT gene families, six and three UGT gene families showed identical and decreased UGT genes, respectively, in *B. rapa* compared with those in *A. thaliana*. For the UGT gene families in *B. oleracea* and *A. thaliana*, 13, five, and two UGT gene families showed increased, identical, and decreased UGT genes, respectively, in *B. oleracea* compared with those in *A. thaliana*, indicating the member expansion of most UGT gene families in *B. oleracea*. By combining the expansion of UGT gene families in *B. rapa* and *B. oleracea*, we obtained that 10 common UGT gene families exhibit increased members of UGT gene family in *B. rapa* and *B. oleracea* compared with *A. thaliana*.

### The effect of WGT and TD events on UGT super gene family

The WGT and TD events engendered the increase in UGT genes in *Brassica* species leading to the expansion of the UGT super gene family. Analysis of retention or loss of UGT genes in UGT gene families in *Brassica* revealed that 10 UGT gene families in *Brassica* show increased gene family members in *Brassica* species. Except for the 10 increased UGT gene families, the remaining UGT gene families were influenced by WGT or TD events; however, not all UGT gene families showed the increase in family members in the *Brassica* species. For UGT genes in *A. thaliana*, 74 of 115 UGT genes were used to detect corresponding UGT genes in *Brassica* species, and 63 UGT genes were generated from the TD event. A total of 63 UGT tandem-duplicated genes were distributed to 10 UGT gene families in *A. thaliana*. For UGT genes in *B. rapa*, 94 and 67 UGT genes were generated from WGT and TD events and were distributed into 18 and 9 UGT gene families, respectively. Out of these UGT gene families, nine UGT gene families were influenced by WGT and TD events; the other nine UGT gene families were influenced by the WGT event, and only one UGT gene family was influenced by the TD event. For UGT genes in *B. oleracea*, 18 UGT gene families including 90 UGT genes were influenced by the WGT event, and nine UGT gene families containing 49 UGT genes were influenced by TD events. From comparison of influence of WGT and TD events, nine UGT gene families experienced WGT and TD events, and the remaining 9 UGT gene families only experienced the WGT event (Table 4).

**Table 4:** Comparison of UGT genes from whole genome triplication and tandem duplication events among *A. thaliana*, *B. rapa*, and *B. oleracea*

Family	*Arabidopsis thaliana*			*Brassica rapa*			*Brassica oleracea*		
	Total UGTs	UGTs from WGT	UGTs from TD	Total UGTs	UGTs from WGT	UGTs from TD	Total UGTs	UGTs from WGT	UGTs from TD
UGT71	14	9	13	20	12	11	20	12	8
UGT72	9	4	2	14	7	4	13	6	2
UGT73	13	8	13	26	14	19	21	12	11
UGT74	7	6	2	11	10	4	14	7	5
UGT75	4	4	0	7	3	2	10	5	4
UGT76	21	15	15	20	15	11	22	16	9
UGT78	4	1	3	1	1	0	1	1	0
UGT79	11	2	4	6	1	4	1	1	0
UGT80	2	2	0	2	2	0	4	4	0
UGT82	1	1	0	2	2	0	2	2	0
UGT83	1	0	0	1	0	0	1	0	0
UGT84	6	6	5	9	8	4	10	8	3
UGT85	6	6	4	9	7	6	9	4	3
UGT86	2	2	0	3	2	0	3	2	0
UGT87	2	0	2	3	0	2	2	0	0
UGT88	1	1	0	1	1	0	1	1	0
UGT89	4	2	0	4	3	0	5	3	4
UGT90	3	3	0	4	4	0	4	4	0
UGT91	3	1	0	3	1	0	3	1	0
UGT92	1	1	0	1	1	0	1	1	0
Total	115	74 (64.35%)	63 (54.78%)	147	94 (63.95%)	67 (45.57%)	147	90 (61.22%)	49 (33.33%)

*Abbreviations*: *WGT* whole genome trplication, *TD* tandem duplicatio

### Expression analysis of UGT genes in *B. rapa, and B. oleracea*

To detect the expression differences of UGT genes in *Brassica* species, we analyzed the transcript abundances of UGT genes in six tissues by using RNA-seq data from NCBI-GEO database [37]. After the transcript abundance of UGT genes in *Brassica* species was filtered, 143 and 141 UGT genes were examined to be expressed across six different tissues in *B. rapa* and *B. oleracea*, respectively. Through hierarchical clustering analysis of expression values of UGT genes in *Brassica* species, the UGT genes were grouped into nine (a, b, c, d, e, f, g, h, and i) and five (j, k, l, m, and n) clusters in *B. rapa* and *B. oleracea*, respectively; this result indicates that UGT genes in *B. rapa* performed more discrete expression patterns than those in *B. oleracea*. In *B. rapa*, most UGT genes were detected to express in one or more tissues. The numbers of expressed UGT genes were relatively higher in silique but lower in leaf compared with other tissues in a UGT gene cluster after excluding the low and missing expression values of UGT genes. In the *b* cluster, most UGT genes were detected to display down-regulated expression levels in root and silique, and up-regulated expression level in callus. In the *d* cluster, the expressed UGT genes showed consistent expression patterns in callus but converse expression patterns in root with expressed UGT genes in *b* cluster. The expressed UGT genes in f, g, h, and i clusters indicate consistent downregulation in callus (Additional file 4A). In *B. oleracea*, the expressed UGT genes in the *j*, *l*, and *m* clusters exhibit upregulated expression in callus; however, these UGT genes from *j*, *l*, and *m* clusters appeared to be downregulated in root, leaf, and flower tissues, respectively. This result indicates that the UGT genes of the *j*, *l*, and *m* clusters were easily reduced by wounding, but these genes also performed tissue-specific expression in *B. oleracea*. Compared with the expression of UGT genes in the *j*, *l*, and *m* clusters, the expressed UGT genes in the *n* cluster showed consistent downregulated expression in callus, indicating that the expression levels of these UGT genes are not easily decreased by wounding. These UGT genes exhibit that same expression patterns as the expressed UGT genes in *f*, *g*, *h*, and *i* clusters in *B. rapa* (Additional file 4B). In general, UGT genes in *Brassica* species exhibit differential expression patterns across different tissues, leading to different functional clusters and suggesting functional divergences. The expressed UGT genes in different functional clusters performed virtually consistent expression patterns in each tissue, suggesting functional conservation. Determining whether the expressed UGT genes indicate functional divergences or conservative will improve the adaptability of changing environment for plants.

### The influence of WGT and TD events on UGT73 gene family in *B. rapa* and *B. oleracea*

**Fig. 6:**
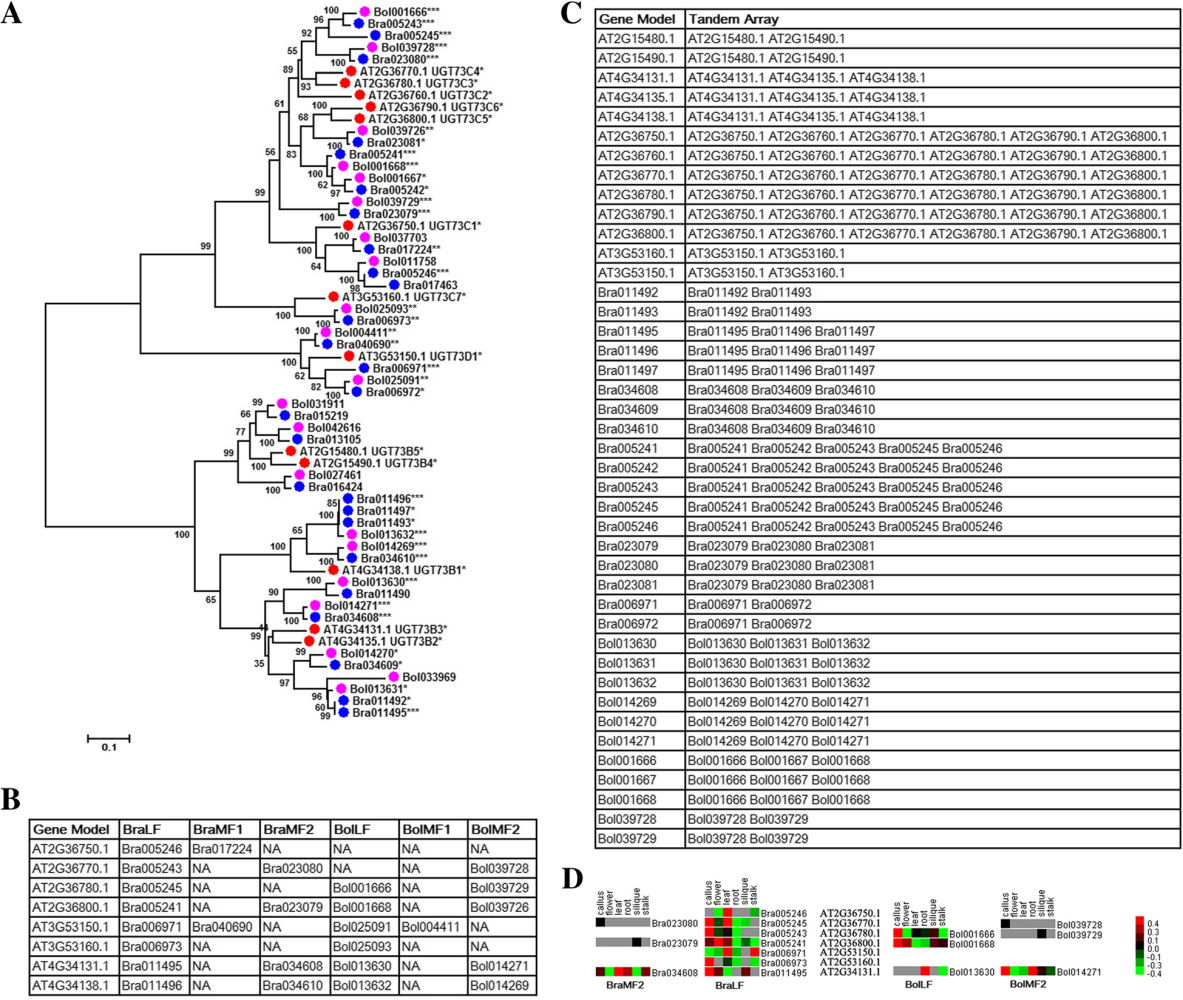
Analysis of expansion mechanism and expression patterns analysis of UGT73 gene family in *A. thaliana*, *B. rapa*, and *B. oleracea*. **a** Phylogenetic relationship of UGT73 gene family in *A. thaliana* and *Brassica* species. Red, blue, and pink solid circles represent the UGT genes in *A. thaliana* and *Brassica* species. **b** UGT tandem-duplicated genes in *A. thaliana* and *Brassica* species. **c** Syntenic orthologous gene pairs between *A. thaliana* and *Brassica* species. **d** The expression analysis of the members of CYP73 gene family from WGT and TD events

By combining the analysis of WGT with TD events of UGT gene families, we selected UGT73 gene family as example to detect the expansion mechanism of UGT gene families in *Brassica* species owing to a relatively large number of the members of UGT73gene family. In the UGT73 gene family, 13 *A. thaliana* UGT genes obtained 26 and 21 UGT homologous genes in *B. rapa* and *B. oleracea*, respectively (Fig. 6a). Among 47 UGT homologous genes, 26 and 29 UGT genes were separately generated from WGT and TD events, meaning that the tandem-duplicated genes are distributed on syntenic genomic regions from WGT event in *Brassica* species. This result suggests that the tandem-duplicated genes located on syntenic genomic regions from WGT event may be generated before the WGT event and implying ancient tandem-duplicated UGT genes in *Brassica* species. From the phylogenetic analysis of UGT gene family among three species, the members of the UGT73B subfamily are clustered into a single subgroup, and the members of UGT73C and UGT73D subfamilies are clustered into the other single subgroup (Fig. 6b). Five UGT genes were identified in the UGT73B subfamily in *A. thaliana* and were detected to be distributed in two tandem arrays in *A. thaliana*. One tandem array contained three UGT genes (AT4G34131.1, AT4G34135.1, and AT4G34138.1, named UGT73B3, UGT73B2, and UGT73B1, respectively) and the other array contained two UGT genes (AT2G15480.1 and AT2G15490.1 named UGT73B5 and UGT73B4, respectively) in *A. thaliana*. For the three-gene tandem array, two UGT genes (AT4G34131.1 and AT4G34138.1) were examined and proven to be two UGT orthologous genes retained in *B. rapa* and *B. oleracea*, respectively, inducing the expansion of the two genes in the UGT73B subfamilies in *Brassica* species (Fig. 6c).

In order to detect the expression influence of WGT and TD events on the members of UGT73 gene family in *B. rapa* and *B. oleracea*, we compared the expression patterns of the members of UGT73 gene family from WGT and TD events respectively (Fig. 6d). For example, one *A. thaliana* tandem array included AT2G36770.1, AT2G36770.1, and AT2G36780.1, which were detected two syntenic tandem arrays in different subgenomes (Bra005245, Bra005243, and Bra005241 in BraLF; Bra023080 and Bra023079 in BraMF2) in *B. rapa*, and two syntenic tandem arrays in different subgenomes (Bol001666, and Bol001668 in BolLF; Bol039728 and Bol039729 in BolMF2) in *B. oleracea*. For the members of CYP73 gene family in *B. rapa*, Bra005245, Bra005243 and Bra005241 in BraLF subgenome were all expressed in the root tissue, and Bra005245 and Bra005243 were expressed in the callues tissue. Theses tandem duplicated genes showed identical expression patterns in certain tissues. Their corresponding orthologous UGTs in BraMF2 subgenome have low expression values or were not detected expression. For the members of CYP73 gene family in *B. oleracea*, Bol001666 and Bol001668 in BolLF subgenome were all expressed in the callues tissue, but their corresponding orthologous UGTs in BolMF2 subgenome have very low expression values or were not detected expression. So, the UGTs from TD events indicated similarity expression patterns but different with that in their corresponding orthologous UGTs from WGT event.

## Discussion

### Emergence of UGT gene families in plants

To trace the evolutionary history of UGT genes, we identified 3, 21, 140, 200, 115, 147, and 147 UGT genes in *C. reinhardtii*, *P. patens*, *S. moellendorffii*, *O. sativa*, *A. thaliana*, *B. rapa*, and *B. oleracea*, respectively. The seven plant species were representative species in algae, bryophytes, pteridophytes, monocots, and dicots, which have completed the genome sequencing. The complete genome sequenced species provided a good opportunity to study the evolution of gene family in plants based genome wide. *C. reinhardtii* is a single-cell green alga and is an especially well studied biological model organism [26]. By using HMM search, we have identified three UGT genes in *C. reinhardtii*. From the phylogenetic analysis of total UGT genes in seven species, we found that only one UGT gene family has been detected to possess corresponding orthologous genes in other six species. These UGT genes were clustered together and named the UGT80 gene family by the nomenclature committee of UGT super gene family, indicating that the members of UGT80 gene family originated from ancestral single-cell aquatic plants. The UGT80 gene family has two members in *A. thaliana*, including UGT80A2 (AT3G07020) and UGT80B1 (AT1G43620). UGT80A2 was investigated to be required for normal levels of major steryl glycosides in seeds, whereas UGT80B1 is involved in accumulation of minor steryl glycosides (SG) and acyl steryl glycosides (ASG) compounds [51]. Phylogenetic analysis revealed that these UGTs played critical accessory roles in the life activities of species and were responsible for specialized functions for distinct classes of SG and ASG molecules in plants. For the known UGT gene families, the members of UGT74 gene family were identified in *S. moellendorffii*, *O. sativa*, *A. thaliana*, *B. rapa*, and *B. oleracea*, indicating that this gene family is shared by ferns and angiosperms or that this gene family appeared after the emergence of ferns. The remaining 17 UGT gene families are shared by angiosperms, meaning that these gene families appeared after the emergence of angiosperms. Moreover, the members (UGT81A1: AT4G31780; UGT81A3: AT5G20410; UGT81A4: AT2G11810) of UGT81 gene family were not classified in *A. thaliana* due to the absent UGTs conserved domain in the three UGTs. Through the analysis of conserved domains of UGT81 gene family, the three members of UGT81 gene family contained mnogalactosyldiacylglycerol (MGDG) synthase (PF06925) and MGDG synthase type C (PF04101) conserved domains, which were involved in the galactolipid biosynthesis [52, 53]. So, we excluded the analysis of UGT81 gene family in UGT super gene family among above seven plant species.

### The TD event occurred continuously throughout the evolutionary history of UGT gene family

Analysis of phylogenetic relationship revealed that *A. thaliana* and *Brassica* ancestor diverged from a common ancestor. According to syntenic relationship in *B. rapa* and *B. oleracea* compared with *A. thaliana*, we detected that the UGT orthologous gene pairs in *Brassica* species compared with *A. thaliana* serve as ancient evolutionary evidence and were inherited from their common ancestor. From comparative analysis of WGT and TD events, we discovered that several UGT genes among three species were distributed on syntenic genomic regions in *B. rapa* and *B. oleracea* compared to *A. thaliana* as tandem arrays. In the *A. thaliana* genome, 12 UGT tandem arrays including 31 UGT genes were detected 13 and 11 UGT tandem arrays, which contain 31 and 24 UGT genes in *B. rapa* and *B. oleracea*, respectively. These results show that 13 and 11 UGT tandem arrays in *B. rapa* and *B. oleracea*, respectively were ancient UGT tandem arrays, which were generated before *A. thaliana* and the *Brassica* ancestor split from a common ancestor. From the analysis of the TD event of UGT genes in *Brassica* species, 67 *B. rapa* and 49 *B. oleracea* UGT genes were generated from TD events. After excluding the ancient UGT genes in *B. rapa* and *B. oleracea*, 36 and 25 UGT tandem-duplicated genes remained in *B. rapa* and *B. oleracea*, respectively*.* These remaining UGT tandem-duplicated genes have not been detected with corresponding orthologous genes in *A. thaliana*, meaning that these genes were generated after the *Brassica* ancestor split from a common ancestor with *A. thaliana*. The remaining UGT tandem-duplicated genes were specific UGT genes in *B. rapa* and *B. oleracea*, respectively. These results suggest that the TD event occurs continuously in the UGT super gene family and is an ongoing process throughout the evolutionary history of the UGT gene family.

### Expansion mechanism of UGT gene families in *B. rapa, and B. oleracea*

Most of the multiple family genes have experienced WGT and TD events, and then the increased genes from the two events undergone retention or loss in evolutionary process. Each multiple family has its specific expansion mechanism. NBS-encoding genes were key plant disease resistance genes, which play an important role in offering resistance to pathogens. The analyses of TD and WGT events of NBS-encoding genes revealed that NBS-encoding homologous gene pairs on triplicated regions in *Brassica* ancestor were deleted or lost quickly, but NBS-encoding genes in *Brassica* species experienced species-specific gene amplification by TD events after divergence of *B. rapa* and *B. oleracea* [54]. By using HMM search, we have identified 147 UGT genes in each of *B. rapa* and *B. oleracea*, approximately 1.3 times the number in *A. thaliana*. For the UGT super gene family in *A. thaliana*, *Brassica* species presented the member expansion of the UGT super gene family compared with *A. thaliana*. The previous study revealed that the WGD and TD events could yield rich genomic material, leading to the expansion of gene family in plants. According to the analysis of the WGD event in *B. rapa*, 94 UGT genes were generated from the WGD event, representing 63.95% of total UGT genes in *B. rapa*. For UGT genes in *B. oleracea*, 90 UGT genes were influenced by the WGD event representing 61.22% of total UGT genes in *B. oleracea.* The proportion of WGD type UGTs in *B. rapa* is slightly higher than that in *B. oleracea*, but they have no significant difference between them (χ2-test, *P* = 0.8171 > 0.05). Through analysis of TD event for UGT gene families in *A. thaliana* and *Brassica* species, 62 *A. thaliana*, 67 *B. rapa*, and 49 *B. oleracea* UGT genes were generated from TD events, accounting for 53.91, 45.58, and 33.33% of all UGT genes in *A. thaliana*, *B. rapa*, and *B. oleracea*, respectively. These results indicate that UGT genes in *A. thaliana* may experience stronger influences from TD event compared with *Brassica* species. From comparisons of influence of WGT and TD events for UGT gene families in *Brassica* species, we can draw a conclusion that WGT event perform more significant effects on the expansion of UGT gene families compared with the TD event in *Brassica* species. The influence of WGT and TD events on UGT gene family revealed that each UGT gene family has its own evolutionary mechanism. Through the expression analysis of UGTs in *B. rapa* and *B. oleracea*, we can see that the UGT genes were grouped into different expression clusters in these two Brassica species, indicating difference of expression patterns of UGTs in *B. rapa* and *B. oleracea*. These results suggested that UGTs in *B. rapa* and *B. oleracea* experienced parallel evolution after they diverged from a common ancestor [55].

## Conclusions

With the development of the plant genome sequencing project, genome release provides a good chance for us to detect the evolutionary history of UGT super gene family in plants. In this study, we have identified 3, 21, 140, 200, 115, 147, and 147 UGT genes in *C. reinhardtii*, *P. patens*, *S. moellendorffii*, *O. sativa*, *A. thaliana*, *B. rapa*, and *B. oleracea* respectively. After phylogenetic analysis of these UGT genes in the seven plant species, we found that the UGT80 gene family is a common ancient gene family that is shared by algae, bryophytes, pteridophytes, and angiosperms, and the UGT74 gene family is shared by ferns and angiosperms, and the remaining UGT gene families are shared by angiosperms. To detect the expansion mechanism and functional characterization of UGT gene family, we systematically investigated the chromosomal distribution, phylogenetic relationship, expansion mechanism, selection pressure, and expression divergence analysis of UGT gene families among *A. thaliana*, *B. rapa*, and *B. oleracea*. Results of chromosomal distribution indicate that 98.6 and 71.4% of UGT genes are located on *B. rapa* and *B. oleracea* pseudo-molecules, respectively. Phylogenetic relationship analysis revealed that UGT genes among three species were classified into three subgroups, which contained three, six, and 12 UGT gene families. Expansion mechanism analyses uncovered that WGT event exerted greater influence than the TD event on the expansion of the UGT super gene family in genus *Brassica*. Analysis of selection forces of UGT orthologous gene pairs in *Brassica* species compared with *A. thaliana* suggests that orthologous genes in *Brassica* species underwent negative selection, but no significant differences were found between *A. thaliana*–*B. rapa* and *A. thaliana*–*B. oleracea* lineages. The emergence of UGT tandem-duplicated genes is continuous and is an ongoing process in the evolutionary history of plants. Our comparisons of the expression divergence of UGT genes between *B. rapa* and *B. oleracea* illustrate that UGT genes in *B. rapa* performed more discrete expression patterns compared with those in *B. oleracea*, indicating stronger function divergence. Combined with phylogeny and expression analysis, the UGTs in *B. rapa* and *B. oleracea* experienced parallel evolution after they diverged from a common ancestor. This work is the first to detect the evolutionary history and functional characterization of UGT super gene family in plants. We hope that our work will provide novel insights into the evolutionary history and functional divergence of special traits or phenotypes of related gene families in plants.

## Additional files

Additional file 1: Chromosomal distribution of UGT genes in *B. rapa* and *B. oleracea.* Green bars represent pseudo-chromosomes in *B. rapa* and *B. oleracea*. A01–A10 represent pseudo-chromosomes in *B. rapa*. C01–C09 represent pseudo-chromosomes in *B. oleracea*. Red rectangles represent UGT gene clusters. (JPEG 2853 kb)

Additional file 2: UGT orthologous gene pairs in *A. thaliana* compared with *B. rapa* and *B. oleracea. (XLSX 13 kb)*

Additional file 3: List of tandem arrays of UGT genes among *A. thaliana, B. rapa*, and *B. oleracea. (XLSX 19 kb)*

Additional file 4: Heat map representation of UGT genes in *B. rapa* and *B. oleracea*. A. Heat map representation of UGT in *B. rapa*. a–i represent the separate functional clusters of UGT genes. B. Heat map representation of UGT genes in *B. oleracea*. j–n represent separate functional clusters of UGT genes. The tissues are shown on the top of each column. The genes are designed on right expression bars. Color scale bars are designed on the top of each heat map. (JPEG 925 kb)

## Abbreviations

HMM: Hidden Markov model
ML: Maximum likelihood
Mya: Million years ago
TD: Tandem duplication
WGD: Whole genome duplication
WGT: Whole genome triplication

## References

[1] Roda F, Walter GM, Nipper R, Ortiz-Barrientos D (2017). Genomic clustering of adaptive loci during parallel evolution of an Australian wildflower. Mol Ecol..

[2] Lamichhaney S, Fuentes-Pardo AP, Rafati N, Ryman N, McCracken GR, Bourne C, Singh R, Ruzzante DE, Andersson L. Parallel adaptive evolution of geographically distant herring populations on both sides of the North Atlantic Ocean. PNAS. 2017;114(17):E3452–E61. 10.1073/pnas.1617728114 PMC5410801 28389569

[3] Herold S, Kuhn M, Bonin MV, Stange T, Platzbecker U, Radke J, Lange T, Sockel K, Gutsche K, Schetelig J, et al. Donor cell leukemia: evidence for multiple preleukemic clones and parallel long term clonal evolution in donor and recipient. Leukemia. 2017. doi:10.1038/leu.2017.104. 10.1038/leu.2017.104 28348390

[4] Sahm A, Bens M, Platzer M, Cellerino A (2017). Parallel evolution of genes controlling mitonuclear balance in short-lived annual fishes. Aging Cell.

[5] Mackenzie PI, Owens IS, Burchell B, Bock KW, Bairoch A, Belanger A (1997). The UDP glycosyltransferase gene superfamily: recommended nomenclature update based on evolutionary divergence. Pharmacogenetics.

[6] Graves JL, Hertweck KL, Phillips MA, Han MV, Cabral LG, Barter TT (2017). Genomics of parallel experimental evolution in drosophila. Mol Biol Evol.

[7] Pozo F, Juste J, Vazquez-Moron S, Aznar-Lopez C, Ibanez C, Garin I (2016). Identification of novel Betaherpesviruses in Iberian bats reveals parallel evolution. Plos One.

[8] Yonekura-Sakakibara K, Hanada K (2011). An evolutionary view of functional diversity in family 1 glycosyltransferases. Plant J.

[9] Lee J, Cho CH, Park SI, Choi JW, Song HS, West JA (2016). Parallel evolution of highly conserved plastid genome architecture in red seaweeds and seed plants. BMC Biol..

[10] Monroe JG, McGovern C, Lasky JR, Grogan K, Beck J, McKay JK (2016). Adaptation to warmer climates by parallel functional evolution of CBF genes in *Arabidopsis thaliana*. Mol Ecol.

[11] Roy SW (2016). How common is parallel Intron gain? Rapid evolution versus independent creation in recently created Introns in daphnia. Mol Biol Evol.

[12] Le Roy J, Huss B, Creach A, Hawkins S, Neutelings G (2016). Glycosylation is a major regulator of Phenylpropanoid availability and biological activity in plants. Front Plant Sci.

[13] Jacquin L, Reader SM, Boniface A, Mateluna J, Patalas I, Perez-Jvostov F (2016). Parallel and nonparallel behavioural evolution in response to parasitism and predation in Trinidadian guppies. J Evol Biol.

[14] Grove CS, Bolli N, Manes N, Varela I, Van't Veer M, Bench A (2017). Rapid parallel acquisition of somatic mutations after NPM1 in acute myeloid leukaemia evolution. Br J Haematol.

[15] Westram AM, Panova M, Galindo J, Butlin RK (2016). Targeted resequencing reveals geographical patterns of differentiation for loci implicated in parallel evolution. Mol Ecol.

[16] Emms DM, Covshoff S, Hibberd JM, Kelly S (2016). Independent and parallel evolution of new genes by Gene duplication in two origins of C4 photosynthesis provides new insight into the mechanism of phloem loading in C4 species. Mol Biol Evol.

[17] Morandin C, Tin MM, Abril S, Gomez C, Pontieri L, Schiott M (2016). Comparative transcriptomics reveals the conserved building blocks involved in parallel evolution of diverse phenotypic traits in ants. Genome Biol..

[18] Initiative TAG (2000). Analysis of the genome sequence of the flowering plant *Arabidopsis thaliana*. Nature.

[19] Wang X, Wang H, Wang J, Sun R, Wu J, Liu S, et al. The genome of the mesopolyploid crop species *Brassica rapa*. Nat Genet. 2011;43(10):1035–9. 10.1038/ng.919 21873998

[20] Liu S, Liu Y, Yang X, Tong C, Edwards D, Parkin IA, et al. The *Brassica oleracea* genome reveals the asymmetrical evolution of polyploid genomes. Nat Commun. 2014;5:3930. 10.1038/ncomms4930 PMC4279128 24852848

[21] Town CD, Cheung F, Maiti R, Crabtree J, Haas BJ, Wortman JR, et al. Comparative genomics of *Brassica oleracea* and *Arabidopsis thaliana* reveal gene loss, fragmentation, and dispersal after polyploidy. Plant Cell. 2006;18(6):1348–59. 10.1105/tpc.106.041665 PMC1475499 16632643

[22] Yang TJ, Kim JS, Kwon SJ, Lim KB, Choi BS, Kim JA, et al. Sequence-level analysis of the diploidization process in the triplicated FLOWERING LOCUS C region of *Brassica rapa*. Plant Cell. 2006;18(6):1339–47. 10.1105/tpc.105.040535 PMC1475497 16632644

[23] Blanc G, Hokamp K, Wolfe KH (2003). A recent polyploidy superimposed on older large-scale duplications in the Arabidopsis genome. Genome Res.

[24] Lysak MA, Koch MA, Pecinka A, Schubert I (2005). Chromosome triplication found across the tribe Brassiceae. Genome Res.

[25] Graham GJ (1995). Tandem genes and clustered genes. J Theor Biol.

[26] Zhang Z, Nie C, Jia Y, Jiang R, Xia H, Lv X (2016). Parallel evolution of Polydactyly traits in Chinese and European chickens. Plos One.

[27] Kozlov KN, Samsonov AM, Samsonova MG (2015). Method of entirely parallel differential evolution for model adaptation in systems biology. Biofizika.

[28] Zlatogursky VV (2016). There and back again: parallel evolution of cell coverings in Centrohelid heliozoans. Protist.

[29] Natrajan RC (2015). Breast cancer heterogeneity: parallel evolution or conscious uncoupling?. J Pathol.

[30] Goodstein DM, Shu S, Howson R, Neupane R, Hayes RD, Fazo J (2012). Phytozome: a comparative platform for green plant genomics. Nucleic Acids Res.

[31] Bailey SF, Rodrigue N, Kassen R (2015). The effect of selection environment on the probability of parallel evolution. Mol Biol Evol.

[32] Huala E, Dickerman AW, Garcia-Hernandez M, Weems D, Reiser L, LaFond F (2001). The Arabidopsis information resource (TAIR): a comprehensive database and web-based information retrieval, analysis, and visualization system for a model plant. Nucleic Acids Res.

[33] Cheng F, Liu S, Wu J, Fang L, Sun S, Liu B (2011). BRAD, the genetics and genomics database for Brassica plants. BMC Plant Biol..

[34] Yu J, Zhao M, Wang X, Tong C, Huang S, Tehrim S, et al. Bolbase: a comprehensive genomics database for *Brassica oleracea*. BMC Genomics. 2013;14:664. 10.1186/1471-2164-14-664 PMC3849793 24079801

[35] Yu J, Ke T, Tehrim S, Sun F, Liao B, Hua W (2015). PTGBase: an integrated database to study tandem duplicated genes in plants. Database (Oxford).

[36] Punta M, Coggill PC, Eberhardt RY, Mistry J, Tate J, Boursnell C (2012). The Pfam protein families database. Nucleic Acids Res.

[37] Machado-Schiaffino G, Kautt AF, Kusche H, Meyer A (2015). Parallel evolution in Ugandan crater lakes: repeated evolution of limnetic body shapes in haplochromine cichlid fish. BMC Evol Biol.

[38] Tong C, Wang X, Yu J, Wu J, Li W, Huang J, et al. Comprehensive analysis of RNA-seq data reveals the complexity of the transcriptome in *Brassica rapa*. BMC Genomics. 2013;14:689. 10.1186/1471-2164-14-689 PMC3853194 24098974

[39] Finn RD, Clements J, Eddy SR (2011). HMMER web server: interactive sequence similarity searching. Nucleic Acids Res.

[40] Bilewitch JP, Ekins M, Hooper J, Degnan SM (2014). Molecular and morphological systematics of the Ellisellidae (Coelenterata: Octocorallia): parallel evolution in a globally distributed family of octocorals. Mol Phylogenet Evol.

[41] Jones P, Binns D, Chang HY, Fraser M, Li W, McAnulla C (2014). InterProScan 5: genome-scale protein function classification. Bioinformatics.

[42] Porcelli I, Reuter M, Pearson BM, Wilhelm T, van Vliet AH (2013). Parallel evolution of genome structure and transcriptional landscape in the Epsilonproteobacteria. BMC Genomics.

[43] Wang GD, Zhai W, Yang HC, Fan RX, Cao X, Zhong L (2013). The genomics of selection in dogs and the parallel evolution between dogs and humans. Nat Commun..

[44] Wang Y, Tang H, Debarry JD, Tan X, Li J, Wang X (2012). MCScanX: a toolkit for detection and evolutionary analysis of gene synteny and collinearity. Nucleic Acids Res.

[45] Ghosh S, Chan CK (2016). Analysis of RNA-Seq data using TopHat and cufflinks. Methods Mol Biol.

[46] Yang Z (2007). PAML 4: phylogenetic analysis by maximum likelihood. Mol Biol Evol.

[47] Chaudhary B, Hovav R, Flagel L, Mittler R, Wendel JF (2009). Parallel expression evolution of oxidative stress-related genes in fiber from wild and domesticated diploid and polyploid cotton (Gossypium). BMC Genomics.

[48] Streisfeld MA, Rausher MD (2009). Genetic changes contributing to the parallel evolution of red floral pigmentation among ipomoea species. New Phytol.

[49] Jafarpour FH, Masharian SR (2009). Temporal evolution of product shock measures in the totally asymmetric simple exclusion process with sublattice-parallel update. Phys Rev E Stat Nonlinear Soft Matter Phys.

[50] Kozak KH, Mendyk RW, Wiens JJ (2009). Can parallel diversification occur in sympatry? Repeated patterns of body-size evolution in coexisting clades of north American salamanders. Evolution.

[51] Stucky DF, Arpin JC, Schrick K (2015). Functional diversification of two UGT80 enzymes required for steryl glucoside synthesis in Arabidopsis. J Exp Bot.

[52] Nakamura Y (2013). Galactolipid biosynthesis in flowers. Bot Stud.

[53] Rocha J, Sarkis J, Thomas A, Pitou L, Radzimanowski J, Audry M (2016). Structural insights and membrane binding properties of MGD1, the major galactolipid synthase in plants. Plant J.

[54] Yu J, Tehrim S, Zhang F, Tong C, Huang J, Cheng X, et al. Genome-wide comparative analysis of NBS-encoding genes between Brassica species and *Arabidopsis thaliana*. BMC Genomics. 2014;15:3. 10.1186/1471-2164-15-3 PMC4008172 24383931

[55] Zhang J, Kumar S (1997). Detection of convergent and parallel evolution at the amino acid sequence level. Mol Biol Evol.

