# Supplementary figures and images for: Genome-wide analysis of UDP-glycosyltransferase super family in Brassica rapa and Brassica oleracea reveals its evolutionary history and functional characterization
Source: BMC Genomics. 2017 Jun 23;18:474. doi: 10.1186/s12864-017-3844-x (PMC5481917; doi:10.1186/s12864-017-3844-x)

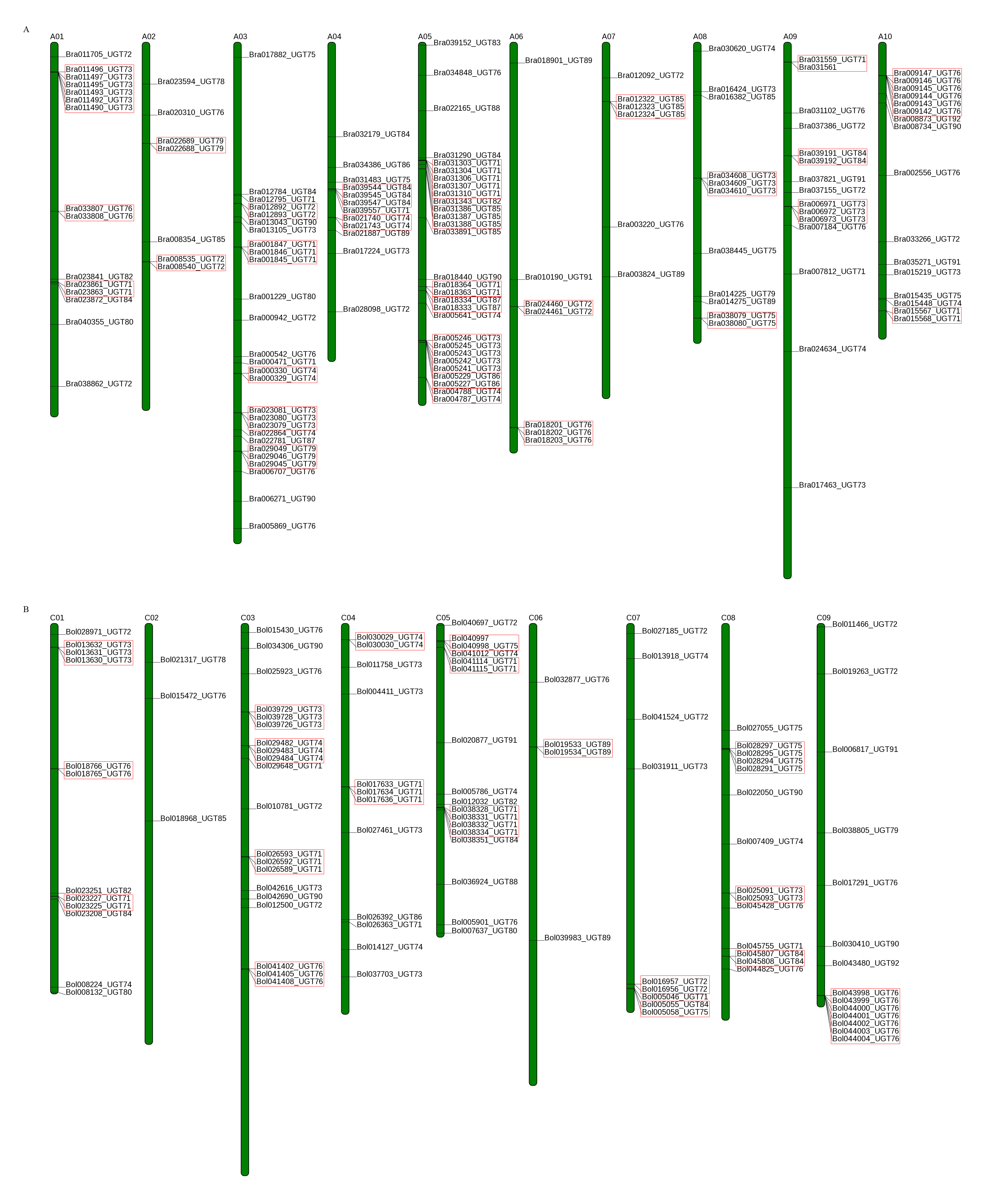

Supplement: Supplementary file 1 — Chromosomal distribution of UGT genes in B. rapa and B. oleracea. Green bars represent pseudo-chromosomes in B. rapa and B. oleracea. A01–A10 represent pseudo-chromosomes in B. rapa. C01–C09 represent pseudo-chromosomes in B. oleracea. Red rectangles represent UGT gene clusters. (JPEG 2853 kb) [file 12864_2017_3844_MOESM1_ESM.jpg]

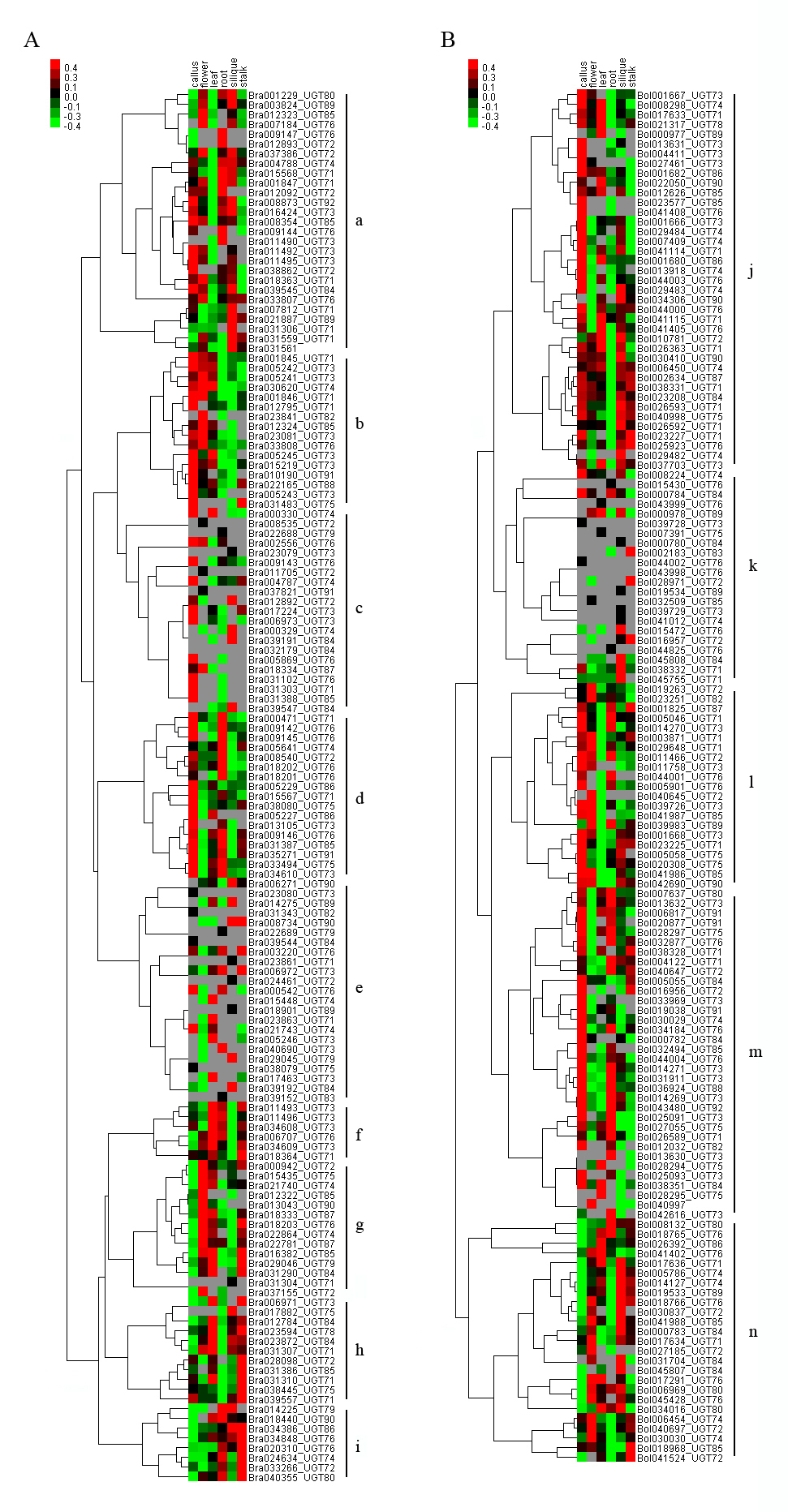

Supplement: Supplementary file 4 — Heat map representation of UGT genes in B. rapa and B. oleracea. A. Heat map representation of UGT in B. rapa. a–i represent the separate functional clusters of UGT genes. B. Heat map representation of UGT genes in B. oleracea. j–n represent separate functional clusters of UGT genes. The tissues are shown on the top of each column. The genes are designed on right expression bars. Color scale bars are designed on the top of each heat map. (JPEG 925 kb) [file 12864_2017_3844_MOESM4_ESM.jpg]
